# Spontaneous acute subdural hematoma as an initial presentation of choriocarcinoma: A case report

**DOI:** 10.1186/1752-1947-2-211

**Published:** 2008-06-19

**Authors:** Brandon G Rocque, Mustafa K Başkaya

**Affiliations:** 1 Department of Neurological Surgery, University of Wisconsin, Madison, WI, USA

## Abstract

**Introduction:**

Diverse sequelae of central nervous system metastasis of choriocarcinoma have been reported, including infarction, intra or extra axial hemorrhages, aneurysm formation and carotid-cavernous fistula. Here we report a case of subdural hematoma as the first presentation of choriocarcinoma.

**Case presentation:**

The patient is a 34-year-old woman whose initial presentation of widely metastatic choriocarcinoma was an acute subdural hematoma, requiring decompressive craniectomy. Histopathologic examination of the tissue showed no evidence of choriocarcinoma, but the patient was found to have diffuse metastatic disease and cerebrospinal fluid indices highly suggestive of intracranial metastasis.

**Conclusion:**

Choriocarcinoma frequently metastasizes intracranially. We review the diverse possible manifestations of this process. In addition, the cerebrospinal fluid:serum beta-human chorionic gonadotropin ratio is an important factor in diagnosing these cases. Finally, the role of the neurosurgeon is discussed.

## Introduction

Choriocarcinoma is a rare gestational trophoblastic disease that complicates approximately 1 in 50,000 term pregnancies and 1 in 30 hydatidiform moles[1]. Among confirmed cases of choriocarcinoma, 45% occur after molar pregnancy, 24% after normal term pregnancy, 25% after spontaneous abortion, and 5% after ectopic pregnancy[2]. Prognosis of this disease is generally good, 80–90% long-term survival with chemotherapy, radiotherapy, and surgical excision in appropriate cases[3]. One of the indicators of a poor prognosis is intracranial metastases, which complicate between 3 and 28% of gestational choriocarcinoma[1]. Here we report a case of subdural hematoma as the first presentation of choriocarcinoma and present a review of the literature pertaining to subdural hematoma in this setting.

## Case Presentation

The patient is a 34-year-old woman who had an acute episode of excruciating headache and was later found obtunded. She had a history of a normal pregnancy three years prior to presentation. She then had an abnormal pregnancy requiring dilation and evacuation at 10–12 weeks that was found to be a molar pregnancy. She became pregnant again 9 months after the dilation and evacuation of the molar pregnancy. This ended in a spontaneous, uncomplicated delivery 5 months prior to her presentation. There was no history of trauma, recent or remote.

**Figure 1:**
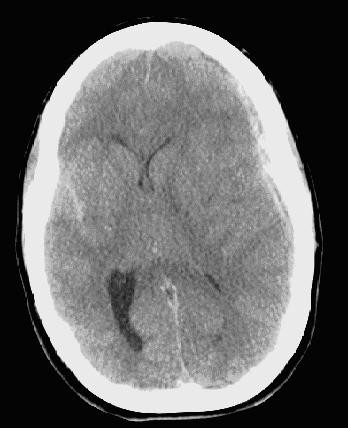
CT scan showing left subdural hematoma with midline shift and right-side subarachnoid hemorrhage.

Upon arrival to Emergency Department, she had fixed, dilated pupils and displayed extensor posturing. Computerized tomography of the head without contrast (Figure 1) showed a 10-mm left hemispheric subdural hematoma causing significant midline shift and uncal herniation. The patient was then taken to the operating room for emergency decompression via frontotemporal craniectomy. A thick, clotted subdural hematoma was removed. Fresh bleeding from one of the cortical arteries was encountered and controlled with bipolar coagulation. Inspection under microscope magnification revealed no obvious vascular or neoplastic lesion. The coagulated part of the small cortical artery was divided and sent for histopathologic examination along with the evacuated hematoma.

Examination of the tissue showed no evidence of vascular malformation or neoplasm, and cytokeratin immunolabeling showed no signs of choriocarcinoma.

**Figure 2:**
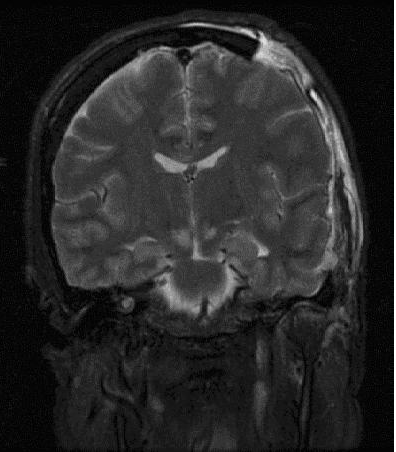
Coronal MRI T2 FLAIR sequence showing herniation injury.

Following neurological and hemodynamic stabilization, CT angiogram showed no evidence of aneurysm or vascular pathology. Magnetic resonance imaging (Figure 2) showed changes associated with herniation injury, but no appreciable tumor or intracranial mass. After full obstetric history was obtained, beta-human chorionic gonadotropin (HCG) level was found to be 55,000 mIU/mL (normal < 5 in non-pregnant patients). CSF examination showed 675 nucleated cells, 20300 red blood cells, protein of 291 mg/dL, glucose of 91 mg/dL, and beta-HCG of 2141 mIU/mL, a serum:CSF ratio of 25:1 (normal > 60:1). CT scan of the chest, abdomen, and pelvis showed lesions in her liver, spleen, kidneys, and lungs. Her neurological status continued to improve. On discharge to the Gynecologic Oncology service one month after presentation, she was extubated and was able to speak slowly, ambulate with assistance, and had no focal motor deficit. She underwent whole brain radiation and chemotherapy with varying regimens of etoposide, cisplatin, bleomycin, methotrexate, cyclophosphamide, and vincristine. She initially did well and was able to transfer to inpatient rehab. However, she developed fibrotic lung disease and then recurrent pulmonary choriocarcinoma lesions, which led to her death four months after her initial presentation.

## Discussion

Approximately one half of tumor-related hemorrhages are the first manifestation of the tumor. In addition, there are numerous reports in the literature of other presentations, including intracranial hemorrhage[4], subarachnoid hemorrhage from rupture of neoplastic aneurysm[5,6], carotid cavernous fistula[7], and infarct due to tumor embolus[8].

Here we report a case of choriocarcinoma presenting as subdural hematoma. This has been reported only twice before in the literature. In 1986, Toyama *et al*. reported a patient who presented with a subdural hematoma due to ruptured aneurysm of the angular artery following surgical resection of a choriocarcinoma in the left adnexa[9]. Histological examination of the tissue confirmed choriocarcinoma in the aneurysm. Cave reported a case of sudden death seven months postpartum due to choriocarcinoma, metastatic to the wall of a ruptured occipital artery[10]. The patient presented with an acute subdural hematoma.

In the female patient of reproductive age, choriocarcinoma must be considered in the differential for any intracranial hemorrhage. A lesion may be apparent on CT scan, but often there is no lesion visible apart from the hemorrhage. Suresh reports a series of 10 hemorrhages from confirmed cases of choriocarcinoma in which only two had visible lesions on CT[11]. The key diagnostic feature, apart from clinical suspicion, is the elevation of beta-HCG in the serum and CSF. Elevated HCG in the serum of a patient with previous abnormal pregnancy strongly suggests choriocarcinoma or retained trophoblastic tissue. If the ratio of serum to CSF HCG is less than 60, CNS metastasis is strongly suspected[12]. The unique feature of the case presented here is the lack of histological confirmation of choriocarcinoma. A diagnostic technique not utilized in this case was serial CSF sampling for beta-HCG. Given the importance of the serum:CSF ratio of beta-HCG in this patient with no other evidence of intracranial disease, serial CSF analysis would allow analysis of the trend as blood is reabsorbed. Presumably, if the decreased serum:CSF ratio is due to contamination with blood from hemorrhage, the ratio would normalize on serial studies. This technique was not utilized in this case, but may be useful in less clear cases. Given her elevated CSF beta-HCG, widespread disease elsewhere, and lack of other factors that could lead to acute subdural hemorrhage, it is clear that the etiology in this case is metastatic choriocarcinoma.

Importantly, CNS metastases are very responsive to chemotherapy. There are reports of complete resolution of CNS disease including intracranial metastases, neoplastic pseudoaneurysms, and neoplastic fistulas with chemotherapy alone [4-7]. Given the good response of this disease to chemotherapy, in many cases, including resolution of CNS pathology, it is not necessary to perform surgical removal of asymptomatic lesions. Surgical treatment should be reserved for patients with symptomatic intracranial pathology that represents an immediate threat.

## Conclusion

Choriocarcinoma is a relatively uncommon malignancy associated with pregnancy. The disease may initially present with intracranial hemorrhage or other CNS manifestation in a significant proportion of patients. It is therefore critical to have a high level of suspicion regarding choriocarcinoma in any patient of reproductive age or with a history of abnormal pregnancy who presents with intracranial pathology. In the case of hemorrhage, it is essential to send the evacuated hematoma for histopathological examination. Increased beta-HCG levels can aid in the diagnosis, and a low serum:CSF beta-HCG level can be strongly suggestive of intracranial choriocarcinoma even in the absence of histopathologically proven disease.

## Consent

Written informed consent was obtained from the family of the patient for publication of this case report and any accompanying images. A copy of the written consent is available for review by the Editor-in-Chief of this journal.

## Competing interests

The authors declare that they have no competing interests.

## Authors' contributions

BGR assembled clinical data and drafted the manuscript, MKB was the primary surgeon and reviewed and revised the manuscript. Both authors read and approved the final manuscript.
